# Macrophage Activation Marker Neopterin: A Candidate Biomarker for Treatment Response and Relapse in Visceral Leishmaniasis

**DOI:** 10.3389/fcimb.2018.00181

**Published:** 2018-06-01

**Authors:** Anke E. Kip, Monique Wasunna, Fabiana Alves, Jan H. M. Schellens, Jos H. Beijnen, Ahmed M. Musa, Eltahir A. G. Khalil, Thomas P. C. Dorlo

**Affiliations:** ^1^Department of Pharmacy & Pharmacology, Antoni van Leeuwenhoek Hospital/the Netherlands Cancer Institute, Amsterdam, Netherlands; ^2^Drug for Neglected Diseases Initiative, Nairobi, Kenya; ^3^Drug for Neglected Diseases Initiative, Geneva, Switzerland; ^4^Division of Pharmacoepidemiology & Clinical Pharmacology, Faculty of Science, Utrecht Institute for Pharmaceutical Sciences, Utrecht University, Utrecht, Netherlands; ^5^Department of Clinical Pharmacology, Antoni van Leeuwenhoek Hospital/the Netherlands Cancer Institute, Amsterdam, Netherlands; ^6^Institute of Endemic Diseases, University of Khartoum, Khartoum, Sudan

**Keywords:** neopterin, biomarker, visceral leishmaniasis, kala-azar, macrophage activation, pharmacodynamics, miltefosine, liposomal amphotericin B

## Abstract

The *Leishmania* parasite resides and replicates within host macrophages during visceral leishmaniasis (VL). This study aimed to evaluate neopterin, a marker of macrophage activation, as possible pharmacodynamic biomarker to monitor VL treatment response and to predict long-term clinical relapse of VL. Following informed consent, 497 plasma samples were collected from East-African VL patients receiving a 28-day miltefosine monotherapy (48 patients) or 11-day combination therapy of miltefosine and liposomal amphotericin B (L-AMB, 48 patients). Neopterin was quantified with ELISA. Values are reported as median (inter-quartile range). Baseline neopterin concentrations were elevated in all VL patients at 98.8 (63.9–135) nmol/L compared to reported levels for healthy controls (<10 nmol/L). During the first treatment week, concentrations remained stable in monotherapy patients (*p* = 0.807), but decreased two-fold compared to baseline in the combination therapy patients (*p* < 0.01). In the combination therapy arm, neopterin concentrations increased significantly 1 day after L-AMB infusion compared to baseline for cured patients [137 (98.5–197) nmol/L, *p* < 0.01], but not for relapsing patients [84.4 (68.9–106) nmol/L, *p* = 0.96]. The neopterin parameter with the highest predictive power for VL relapse was a higher than 8% neopterin concentration increase between end of treatment and day 60 follow-up (ROC AUC 0.84), with a 93% sensitivity and 65% specificity. In conclusion, the identified neopterin parameter could be a potentially useful surrogate endpoint to identify patients in clinical trials at risk of relapse earlier during follow-up, possibly in a panel of biomarkers to increase its specificity.

## Introduction

Visceral leishmaniasis (VL) is a systemic disease caused by the *Leishmania* parasite. VL is affecting mostly the poorest of the poor and remains a devastating neglected tropical disease with high morbidity and mortality, with over 200,000 new cases and over 20,000 deaths annually (Alvar et al., 2012). New efficacious, affordable and safe treatments for this devastating disease are urgently needed.

Parasite recrudescence and clinical relapse occur in a relatively large proportion of VL patients (Collin et al., 2004; Sundar et al., 2012; Rijal et al., 2013). In a recently published clinical trial in Kenya and Sudan, 6–18% of patients relapsed within 6 months follow-up, depending on the treatment arm (Wasunna et al., 2016). As parasite recrudescence and clinical relapse is a long-term event, the follow-up period to determine efficacy of new VL treatment regimens is normally 6 or even 12 months. To speed up the process of assessing the efficacy of new treatment regimens, sensitive and specific early biomarkers are required to predict long-term clinical outcomes, e.g., to be used in an adaptive trial design with interim analysis. As yet, no longitudinal evaluations of pharmacodynamic markers have been performed in the evaluation of anti-leishmanial therapies (Kip et al., 2015).

The *Leishmania* parasite resides and replicates within macrophages. In experimental models, effective control of VL infection is related to the activation of macrophages by interferon-γ (IFN-γ) to produce free radicals that kill the intracellular *Leishmania donovani* parasites. *L. donovani* infection causes an increase in monocyte load in the infected organs (Murray et al., 1987; Cervia et al., 1993). The influx of immature macrophages is required to increase the capacity of macrophages to respond to IFN-γ (Murray et al., 1987; Cervia et al., 1993). As the macrophage biomass is increasing in active VL and subsequently decreases again when the parasitic infection is cleared, we hypothesized that a macrophage activation marker could potentially serve as a potential surrogate biomarker to monitor treatment response in VL.

The macrophage activation marker neopterin, a pteridine biosynthesized from guanosine triphosphate, is excreted by activated macrophages/monocytes and its production mirrors the activation of cellular immunity (Murr et al., 2002). The main stimulus for neopterin production is the pro-inflammatory IFN-γ released after T-lymphocyte activation (reviewed by Hamerlinck, 1999; Hoffmann et al., 2003). In theory, neopterin release would rise in VL due to macrophage activation and increase in macrophage load during active disease, and subsequently decrease with waning parasitic infection. After its synthesis, neopterin is metabolically stable and excreted via the kidneys by both glomerular filtration and tubular secretion, with a total clearance of 499 ± 79.7 mL/min (Estelberger et al., 1993).

Average European healthy control neopterin levels (±SD) are 6.78 ± 3.6 nmol/L (*n* = 263) and 5.34 ± 2.7 nmol/L (*n* = 359) for children (<18 years) and adults, respectively (Werner et al., 1987). In general, 10 nmol/L is taken as the upper limit of normal for healthy controls. Given that neopterin is released upon macrophage activation, increased neopterin levels are associated with a variety of conditions involving cellular mediated immunity, such as intracellular bacterial infections (tuberculosis, leprosy), parasites (malaria), and more (reviewed by Hamerlinck, 1999). Pre-treatment neopterin levels in VL patients were previously found to be significantly elevated compared to healthy controls with mean concentrations of 32 nmol/L in patients from the *L. chagasi* VL-endemic region Bahia in Brazil and 40 nmol/L in Dutch and Kenyan VL patients (Schriefer et al., 1995; Hamerlinck et al., 2000). Successful antimonial treatment significantly decreased neopterin levels to healthy control-levels in treatment responders at 30 days post-treatment, but not in refractory patients (Schriefer et al., 1995).

The aim of this study was to further evaluate the potential of neopterin as a reliable predictive biomarker for prediction of treatment outcome in VL in a larger patient population by longitudinal neopterin measurements during and up to 6 months after treatment. In addition, the objective of this study was to characterize the differences in neopterin kinetics over time between the treatment arms in this study: the miltefosine monotherapy and combination therapy of liposomal amphotericin B (L-AMB) and miltefosine.

## Methods

### Ethics, study design, and clinical sample collection

Neopterin concentrations were determined as part of a randomized multicentre trial (registered as NCT01067443) assessing the safety and efficacy of different VL treatments in Eastern Africa. The clinical results and pharmacokinetic analysis have been published elsewhere (Wasunna et al., 2016; Dorlo et al., 2017). The study was carried out at three VL treatment centers located in endemic areas: two in Sudan (Dooka and Kassab hospitals) and one in Kenya (Kimalel health center).

The study was approved by the national and local Ethics Committees in Kenya (Kenya Medical Research Institute) and Sudan (Institute of Endemic Diseases) prior to the start of the trial in each country (Wasunna et al., 2016). In addition, ethical approval was granted by the LSHTM's (London School of Hygiene and Tropical Medicine) Ethics Committee (#5543) and a “declaration of no objection” was issued by the Amsterdam Academic Medical Center Medical Ethics Committee. The study was explained to all subjects or parents/guardians in their own language. Written informed consent, or ascent in the case of minors, was obtained from all participants before enrollment in the study.

Eligible patients were primary VL cases with parasitological confirmation of VL, aged between 7 and 60 years, HIV negative, and without concomitant severe infection or co-morbidities. Parasitological assessment was performed by microscopy on lymph node aspirates (Dooka, Kassab), spleen aspirates (Kimalel), or bone marrow samples (all sites). Samples originated from patients receiving either a 28-day 2.5 mg/kg/day miltefosine monotherapy (48 patients), or a combination treatment of one dose 10 mg/kg L-AMB on day 1 of treatment, followed by a 10-day 2.5 mg/kg/day miltefosine treatment (48 patients).

Patients that required rescue treatment during treatment or patients who had a fatal outcome before the end of treatment were indicated as “initial treatment failure.” Final cure was determined at 6 months after end of treatment (day 210). Patients indicated as “relapse” were cured at the end of treatment, but received rescue treatment within 6 months after treatment due to reappearance of VL clinical signs and symptoms and parasite recrudescence confirmed by microscopy.

To decrease the invasiveness of sampling for patients, neopterin concentrations were quantified in the same samples collected for miltefosine pharmacokinetic analysis (Wasunna et al., 2016; Dorlo et al., 2017). For this reason, baseline samples were taken on the first day of miltefosine treatment before the first miltefosine dose, which in the combination therapy was 1 day after the L-AMB infusion (study day 2). Real baseline neopterin concentrations were thus only available in the miltefosine monotherapy treatment arm, but were assumed to be equal in the combination therapy arm, since patients were randomized and were balanced with respect to baseline characteristics (Wasunna et al., 2016). Further sampling was performed on study days 4, 7, and 11 (combination therapy), or study days 3, 7, 14, and 28 (monotherapy). Both groups had two samples collected during follow-up at one (day 60) and 6 months (day 210) post-treatment. Plasma was collected from sodium heparin whole blood. Samples were stored and transported at nominally −20°C until analysis.

### Analytical method

Neopterin was determined in patient plasma samples with a commercially available ELISA kit (Demeditec, Kiel-Wellsee, Germany), following the manufacturer's instructions. Two calibration curves (0, 1.35, 4.0, 12.0, 37.0, 111 nmol/L) were included in every analysis together with two quality control samples in duplicate. Samples above the upper limit of quantitation were reanalyzed in a 10x dilution with a dilution buffer provided by the manufacturer. The optical density (OD) was measured at 450 nm by an Infinite® M200 Microplate Reader (Tecan, Männedorf, Switzerland). The OD values were converted to neopterin concentrations from the standard curve using a 4 parameter non-linear logistic regression model in Prism (version 6.0, GraphPad, La Jolla, CA, USA).

Incurred sample reanalysis was performed for 4% of all samples. The acceptance criterion was adapted from FDA guidelines for bioanalytical method validation, and stated that at least two-thirds of the analyzed concentrations should be within 20% deviation of the initially analyzed concentration (US Food and Drug Administration FDA, 2001).

Neopterin plasma stability at −20°C was reported to be at least 6 months (in ELISA kit). As incurred sample reanalysis was performed >1.5 years after initial analysis for a proportion of samples, these results were used to assess the influence of long-term storage on neopterin quantification.

### Statistical analysis

Data cleaning and interpretation was performed with R (version 3.1.2) and packages “ggplot2,” “Hmisc,” and “plyr.” All values are reported as the median (IQR, interquartile range). In the display of results, nominal time points are depicted instead of actual time points.

Absolute neopterin concentrations and relative concentration changes over time—during and after treatment—were evaluated for their ability to reliably discriminate between cured and relapsed patients. In statistical comparisons, absolute and log-transformed data were checked for normality and equal variances. The two-sided *t*-test on log-transformed data was used when comparing groups, unless indicated otherwise.

Subsequently, a logistic regression was performed in R to evaluate the significance of the evaluated neopterin parameter as a predictor of clinical outcome. Finally, receiver-operating characteristic (ROC) curves were generated with the R package “pROC.” The interplay between sensitivity and specificity of neopterin as biomarker in isolation was interpreted and the optimal cut-off value was determined with the same package.

## Results

### Patient population, samples, and quality control

A total of 497 plasma samples were available from 96 patients; 48 patients in combination therapy and 48 patients in monotherapy. In both treatment arms, two patients experienced initial treatment failure. Six patients in the combination therapy arm and nine patients in the monotherapy arm relapsed within 6 months after treatment. Of patients experiencing treatment failure, samples were only included up to the day they received rescue treatment; subsequent samples were omitted (*n* = 4).

Patient characteristics are depicted in Table 1. Age distribution and gender ratio were comparable between the two treatment arms. Relapsing patients (*n* = 15) received rescue treatment at day 112 (median, range day 63–217), approximately 3 months after treatment.

**Table 1:** Demographics of patients included in neopterin analysis.

**Parameter**	**Combination therapy arm**	**Monotherapy arm**	**Both arms**	**Significance**
Total no. of patients	48	48	96	n.s.^a^
Female patients [no. (%)]	6 (12.5)	7 (14.6)	13 (13.5)	n.s.^a^
Pediatric patients (≤12 year) [no. (%)]	26 (54.2)	21 (43.8)	47 (49.0)	n.s.^a^
Age (yr)	14 (7–30)	15 (7–41)	15 (7–41)	n.s.^b^
Body weight (kg)	35 (15–59)	37 (16–65)	36 (15–65)	n.s.^b^
**TREATMENT OUTCOME**				
Patients with initial failure [no. (%)]	2 (4.2)	2 (4.2)	4 (4.2)	n.s.^c^
Patients with relapse [no. (%)]	6 (12.5)	9 (18.8)	15 (15.6)	
Patients that cure [no.(%)]	40 (83.3)	37 (77.1)	77 (80.2)	
**TREATMENT CENTERS**				
Kimalel, Kenya [no. (%)]	25 (52.1)	24 (50.0)	49 (51.0)	n.s.^c^
Kassab, Sudan [no. (%)]	6 (12.5)	7 (14.6)	13 (13.5)	
Dooka, Sudan [no. (%)]	17 (35.4)	17 (35.4)	34 (35.4)	

*All values are given as median (range), unless stated otherwise*.

a *Fisher exact test*.

b *Wilcoxon u-test*.

c *Chi-square test*.

During treatment, all actual sample collection time points were within ±15% of the nominal time points. During follow-up, the variability in actual sample collection time points was larger, with day 60 samples collected between day 54–157 and day 210 samples between day 185–345. Nonetheless, >85% of samples were collected within ±15% of the nominal time point during follow-up.

For all runs, quality control samples were within the acceptable range according to ELISA kit specifications. Incurred sample reanalysis was found to be acceptable (>95% of reanalyzed samples were within ±20% deviation of original concentration). Incurred sample reanalysis was also acceptable for the subset (*n* = 12) of samples analyzed >1.5 years after initial analysis (11 out of 12 within ±20% deviation). This indicates adequate stability of neopterin in plasma for at least 1.5 years when stored at −20°C.

### Baseline neopterin concentrations

**Figure 1:**
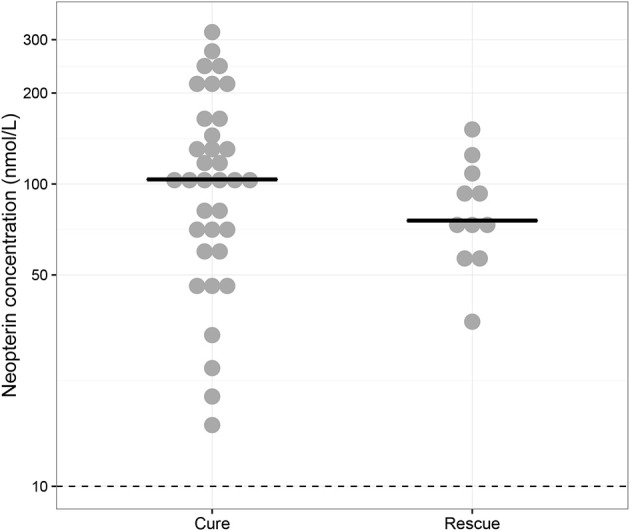
Baseline neopterin concentrations. Individual baseline neopterin concentrations (median indicated with horizontal line) in the monotherapy treatment arm—for which baseline samples were available—stratified for patients that were cured (“Cure”, *n* = 35) and patients that received rescue treatment during or within 6 months after treatment (“Rescue”, *n* = 11). The dotted line indicates the reported upper limit of normal in healthy controls (10 nmol/L).

Baseline neopterin concentrations were elevated in all monotherapy VL patients (*n* = 46) at 98.8 nmol/L (IQR 63.9–135) (Figure 1). There was a non-significant (*p* = 0.448) trend toward higher neopterin baseline levels in monotherapy patients cured at the end of treatment (104 nmol/L, IQR 64.9–154) compared to patients requiring rescue therapy during or within 6 months after treatment (75.7 nmol/L, IQR 65.4–102). There were no significant differences in baseline neopterin levels between age categories (adult/child), country and gender (*p* = 0.955, *p* = 0.620, *p* = 0.737, respectively).

### Neopterin kinetics over time

**Figure 2:**
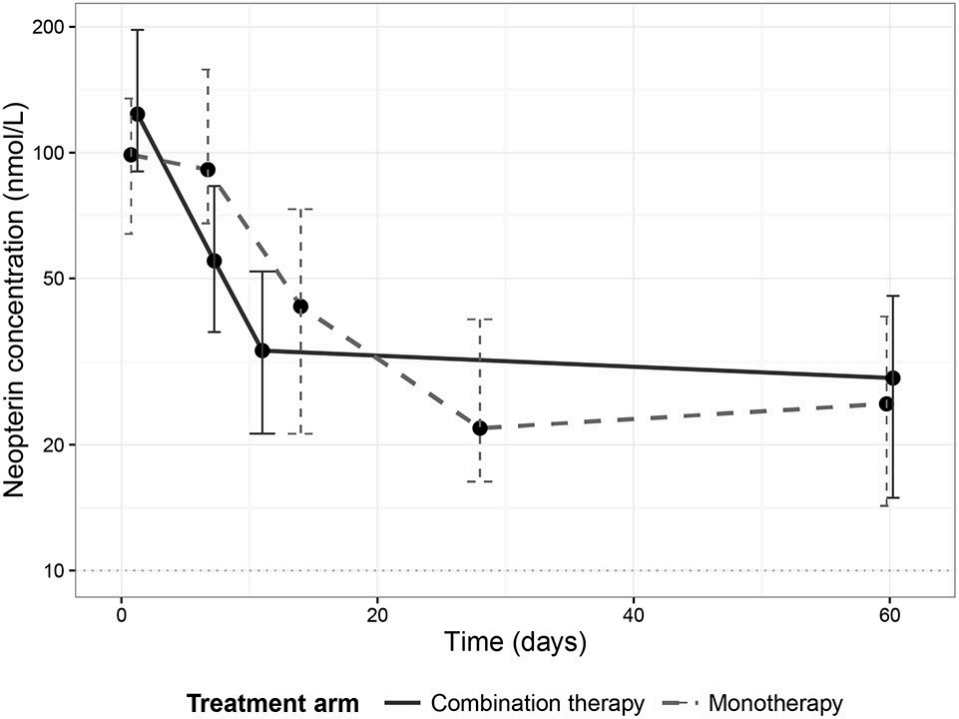
Neopterin dynamics per treatment arm. Dynamics of median neopterin concentrations in visceral leishmaniasis patients undergoing a combination therapy of L-AMB and miltefosine (solid line) or miltefosine monotherapy (dashed line). Error bars represent the inter-quartile range (IQR).

Neopterin concentrations regressed during treatment to comparable end of treatment values of 33.6 nmol/L (IQR 21.3–52.0, combination therapy, day 11) and 21.9 nmol/L (IQR 16.3–40.0, monotherapy, day 28). There was, however, a difference in the rate of neopterin decline between the two treatment arms (Figure 2). Neopterin concentrations decreased two-fold compared to baseline within the first seven treatment days in the combination therapy arm to 55.1 nmol/L (IQR 37.2–83.2, *p* < 0.01), while neopterin levels remained unchanged in patients receiving monotherapy with a concentration of 91.3 nmol/L (IQR 65.9–158, *p* = 0.807, Mann-Whitney *U*-test). Interestingly, for both treatment arms, day 210 neopterin concentrations were still elevated (15.5 nmol/L IQR 10.5–22.3, combination therapy, 13.5 nmol/L IQR 11.4–22.9, monotherapy) compared to the reported healthy control levels of <10 nmol/L.

### Predictive value of absolute neopterin levels for treatment outcome

**Figure 3:**
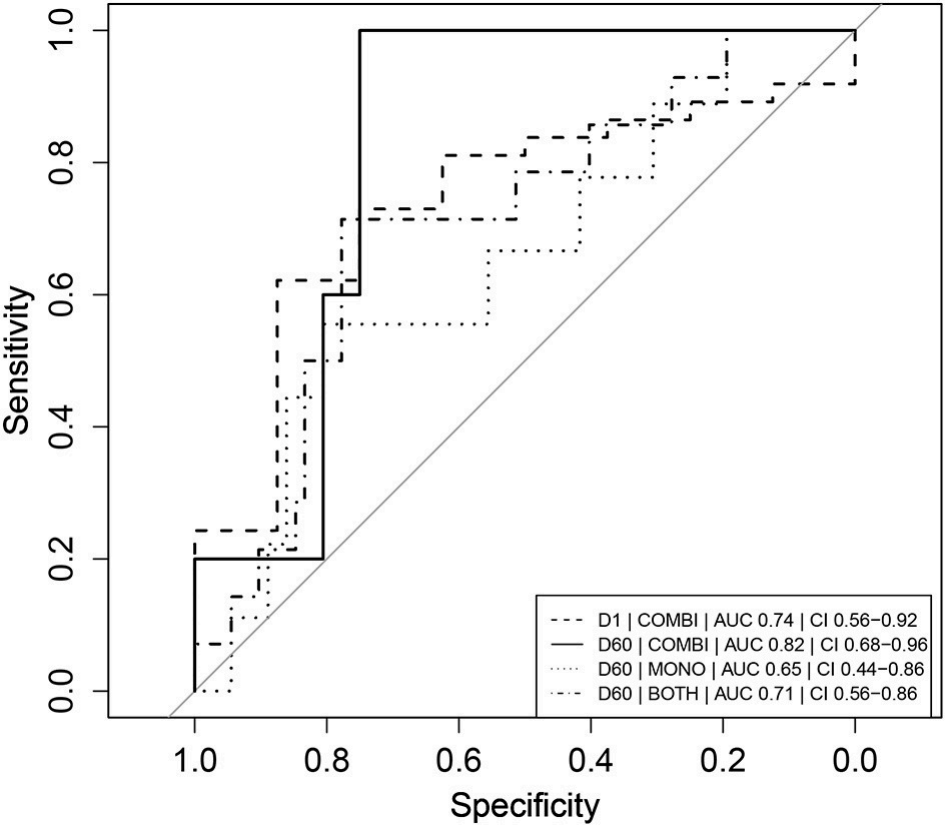
Receiver operator characteristic (ROC) curves of absolute neopterin concentrations as predictors of clinical relapse. Combination therapy is indicated as “COMBI,” monotherapy as “MONO” and data of the two arms combined as “BOTH.” AUC represents the integrated area under the ROC curve. CI refers to the confidence interval of the calculated AUC. Note that day 1 (D1) neopterin concentrations are evaluated as predictor of cure (cure = 1, relapse = 0) and day 60 (D60) neopterin concentrations are evaluated as predictor of relapse (relapse = 1, cure = 0).

One day after L-AMB infusion and before the first miltefosine dose, neopterin concentrations in the combination therapy arm were significantly higher (137 nmol/L, IQR 98.5–197, Table 2) in cured patients, compared to the baseline concentration of 98.8 nmol/L in the monotherapy arm (*p* < 0.01). This was not observed for combination therapy patients who eventually failed treatment or relapsed (84.4 nmol/L, IQR 68.9–106, *p* = 0.96). Despite the significantly higher day 2 neopterin concentrations in cured patients, this parameter is not a significant predictor of final cure (*p* = 0.0853). The ROC AUC (Figure 3) was 0.74 (CI 0.56–0.92) with a low sensitivity (62%, optimal cut-off value of 122 nmol/L).

**Table 2:** Median neopterin concentration split per treatment arm and treatment outcome.

	**Cure**			**Relapse**			
	***N***	**Median neopterin conc. (nmol/L)**	**IQR**	***N***	**Median neopterin conc. (nmol/L)**	**IQR**	***p*-value**
**COMBINATION THERAPY**							
Day 2	37	136.6	98.5–197.2	8	84.4	68.9-105.8	0.05395^a^
Day 4	12	123.9	60.2–304.8	2	84.2	83.6–84.7	0.5495^b^
Day 7	36	58.2	38.8–95.1	6	37.7	33.1–49.6	0.1268^a^
Day 11^**^	36	35.0	25.4–53.3	7	28.1	19.4–38.3	0.3424^a^
Day 60	36	26.3	14.7–40.2	5	54.0	42.0–69.4	0.01969^a^^*^
Day 210	29	16.9	12.0-23.0	5	15.4	12.1–17.5	0.8223^a^
**MONOTHERAPY**							
Day 1	35	103.6	64.9–153.8	11	75.7	65.4–102.0	0.448^a^
Day 3	14	111.3	84.6–156.4	1	32.0	N/A	0.2667^b^
Day 7	36	93.7	75.8–161.7	9	77.8	60.1–135.2	0.2928^b^
Day 14	34	43.5	28.6–68.1	10	33.3	20.6–116.8	0.9293^c^
Day 28^**^	35	22.1	16.5–35.9	10	21.2	14.1–42.5	0.5448^b^
Day 60	36	23.9	14.2–37.6	9	40.6	19.1–61.6	0.1823^a^
Day 210	30	13.5	12.0–22.5	5	10.7	9.4–72.4	0.9091^b^

a *Two-sample t-test on log-transformed neopterin concentrations*.

b *Wilcoxon U-test on absolute neopterin concentrations*.

c *Welch Two-sample t-test on log-transformed neopterin concentrations with unequal variance*.

* *p < 0.05*.

** *End of treatment*.

In the combination treatment arm, patients that relapsed had a significantly higher day 60 neopterin concentration compared to cured patients (54.0 vs. 26.3 nmol/L, *p* < 0.05, Table 2). The same trend was observed for the monotherapy treatment arm, though not significant (Table 2).

The day 60 neopterin concentration was a significant predictor of relapse in both arms combined and in the combination therapy arm (*p* < 0.05), but not for the monotherapy arm alone. ROC curves of these parameters are depicted in Figure 3. In the combination therapy arm, the day 60 neopterin concentration was the best predictor of relapse with an AUC of 0.82 (CI 0.68–0.96) and optimal threshold value of 39.7 nmol/L with corresponding sensitivity of 100% and specificity of 75%. In the monotherapy arm, the absolute neopterin concentration at day 60 was a less reliable predictor of relapse (AUC 0.65), with a sensitivity of only 56% (at optimal cut-off 40.2 nmol/L, 81% specificity).

### Predictive value of relative neopterin levels for treatment outcome

**Figure 4:**
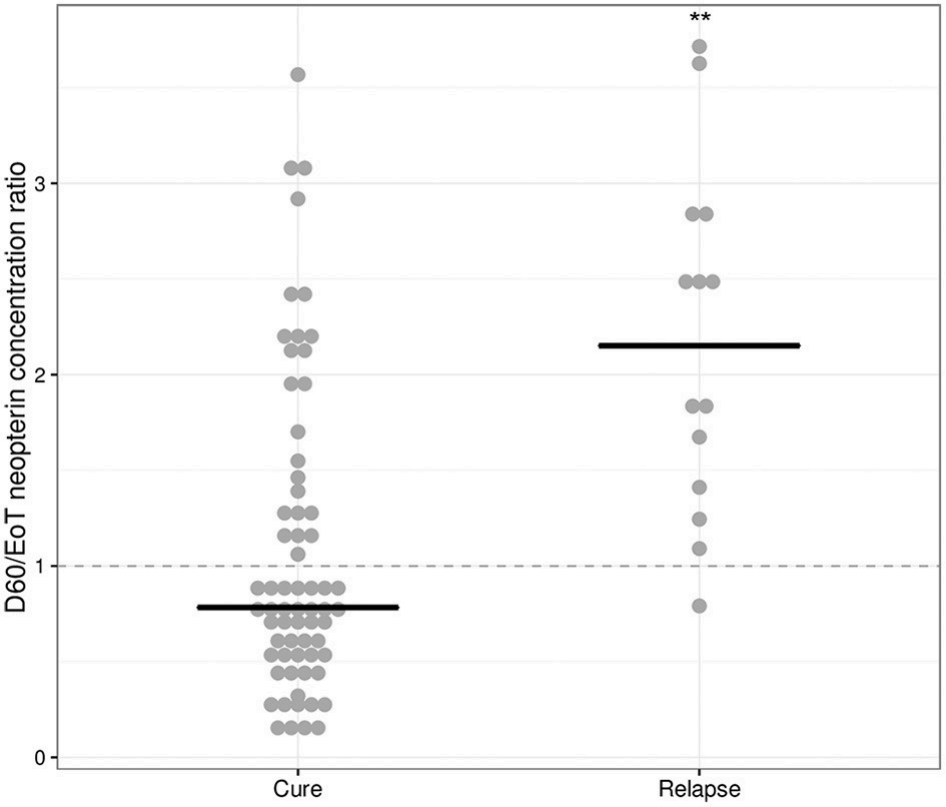
D60/EoT neopterin concentration ratio for cured and relapsed patients. D60/EoT neopterin concentration ratio, for cured patients (*n* = 66) and patients that relapsed after treatment (*n* = 14). The dashed line indicates no difference within 1 month after end of treatment (combination therapy: day 11, monotherapy: day 28). Dots indicate individual observations, the horizontal lines the median per group. ^**^p < 0.001, Welch *t*-test on log-transformed data.

An increase in neopterin concentrations was observed for relapsing patients between end of treatment and day 60 (Table 2), but not for cured patients. The D60/EoT neopterin concentration ratio (or D60/EoT ratio)—a patient's neopterin concentration on day 60 relative to the end of treatment (EoT) concentration—could be calculated for 80 patients and is shown in Figure 4. Relapsing patients (*n* = 14) experienced a significantly higher neopterin concentration increase during the first month of follow-up (D60/EoT ratio: 2.2, IQR 1.5–2.8) compared to patients that remained cured (D60/EoT ratio: 0.78, IQR 0.53–1.4) (*p* < 0.001, Welch *t*-test on log**-**transformed data). For patients that relapsed, there was no correlation between the D60/EoT ratio and the day they received rescue treatment (linear regression *R*^2^ = −0.009).

**Figure 5:**
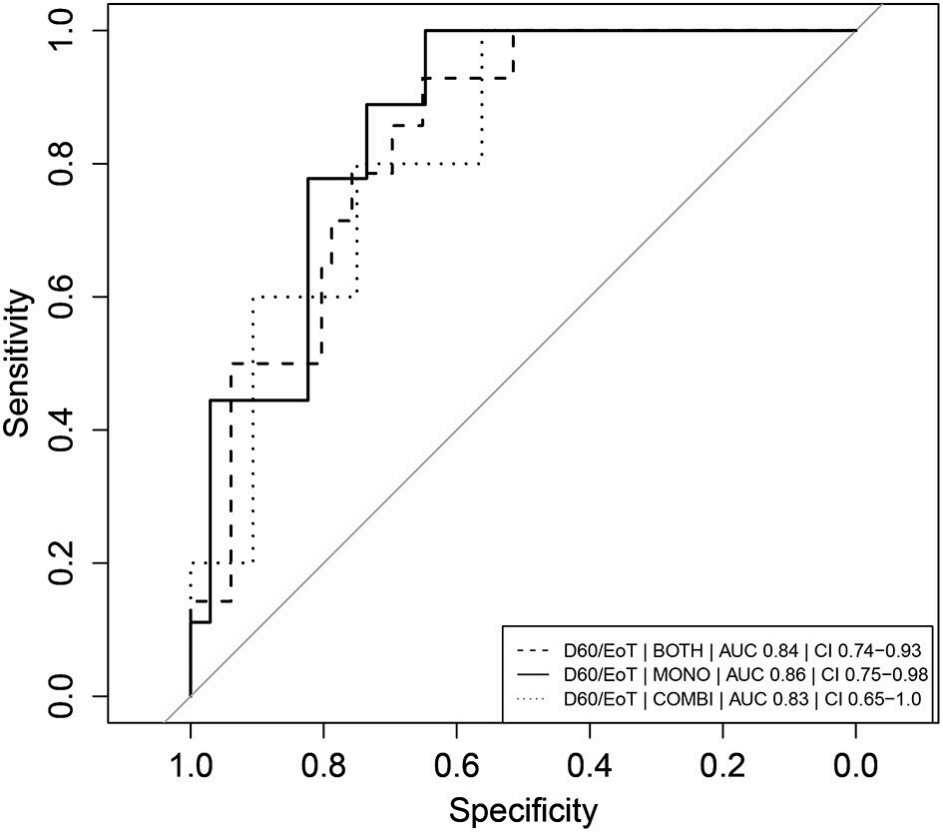
Receiver operator characteristic (ROC) curves of D60/EoT neopterin concentration ratio as predictor of clinical relapse. Receiver operator characteristic (ROC) curves of D60/EoT neopterin concentration ratio as predictor for treatment failure (relapse = 1, cure = 0). Combination therapy is indicated as “COMBI,” monotherapy as “MONO” and data of the two arms combined as “BOTH.” AUC represents the integrated area under the ROC curve. CI refers to the confidence interval of the calculated AUC.

The D60/EoT neopterin concentration ratio was a significant predictor of relapse for both arms combined (*p* < 0.001), the monotherapy (*p* < 0.01), and combination therapy (*p* < 0.05) separately. ROC curves are depicted in Figure 5. With a cut-off of 1.08, the D60/EoT ratio neopterin parameter had a sensitivity of 93% and specificity of 65% in predicting relapse (ROC AUC 0.84).

## Discussion

This study is the first longitudinal exploration of the kinetics of neopterin in VL patients, before, during and after treatment, to identify a predictive host-related biomarker for the long-term treatment outcome and relapse in VL.

We identified several neopterin parameters that could potentially be used as early predictors of clinical relapse, evaluated based on their ROC AUC. As a general rule, AUCs of 0.7–0.8 are considered acceptable, 0.8–0.9 excellent and >0.9 outstanding (Hosmer and Lemeshow, 2000). The D60/EoT neopterin concentration ratio was found to be a significant and highly sensitive predictor of relapse at a cut-off of 1.08 (AUC 0.84).

As a subset of cured patients also demonstrated an increase in neopterin concentration after end of treatment, specificity was relatively low. No clinical explanation could be identified for the neopterin concentration increase on day 60 in these cured patients: there were no consistent trends in fever, hematological or clinical chemistry parameters, nor were there more co-infections or concomittant medications reported in these patients. An additional limitation of the study was the relatively small group of relapses (*n* = 14), which might have impeded statistical power to detect predictors for relapse.

Although increased neopterin concentrations have also been observed for other infectious diseases (Murr et al., 2002), the observed baseline neopterin concentration in this study was substantially higher than observed in other diseases, such as HIV (17–50 nmol/L) (Fuchs et al., 1990), tuberculosis (21–37 nmol/L) (Hosp et al., 1997; Cesur et al., 2014; Skogmar et al., 2015), and malaria (21–58 nmol/L) (Thuma et al., 1996; Biemba et al., 2000). It should be noted that the observed baseline concentrations in this study were higher than previously reported for VL patients (Schriefer et al., 1995; Hamerlinck et al., 2000), possibly due to different causative *Leishmania* subspecies or severity of disease. D60/EoT ratio might be more prone to specificity issues in co-infection, since concentrations are lower at those time points.

Depending on the purpose of use, the minimally acceptable characteristics of pharmacodynamic biomarkers concerning specificity will differ. Requirements are less strict in a clinical trial setting, as concomitant disease is often an exclusion criteria. Another solution for lack of specificity could be to use a panel of biomarkers. In routine clinical care, the implementation of the D60/EoT ratio and corresponding sampling point 1 month after treatment, is probably problematic due to the remote and/or resource-poor settings.

An advantage of neopterin as a pharmacodynamic biomarker is the relatively low cost of analysis at around 3 euro per clinical sample for a commercial kit. Nevertheless, a basic laboratory infrastructure is required, which is not always available in health centers in the resource-limited regions where VL is being treated. A simple dipstick assay is available for the semi-quantitative detection of neopterin in serum and has also been tested in VL patients (Bührer-Sekula et al., 2000), though this assay is possibly not sensitive enough to detect the relatively subtle concentration changes after treatment. Easier, cheaper and less invasive neopterin analytical methods have been developed in dried blood spots and urine, but these have not yet been evaluated in VL patients (Zurflüh et al., 2005; Svoboda et al., 2008; Opladen et al., 2011).

This study also explored differences in neopterin kinetics between treatment regimens in patients treated with either miltefosine monotherapy or miltefosine in combination with L-AMB. This longitudinal analysis revealed a different neopterin kinetic profile for the two treatment arms, possibly implying a difference in the elicited immune reaction. The initial surge in neopterin levels within 1 day after L-AMB infusion in cured patients could suggest a beneficial effect of early activation of the pro-inflammatory T_h_1 response initiated by the L-AMB infusion. A significant similar rise in pro-inflammatory cytokines was also observed in mice with *Aspergillus flavus* infection treated with L-AMB (Olson et al., 2012). Further clinical research is needed to confirm these findings and further investigate the underlying mechanisms, but one possible explanation could be that L-AMB positively reinforces and amplifies already persisting immune reactions. The stable neopterin concentrations in the first week of miltefosine monotherapy correlate with the continuous slow accumulation of the drug and potentially less effective exposure in the early phase of treatment.

In both treatment arms, neopterin concentrations were still elevated 6 months post-treatment in comparison to the reported healthy control value of <10 nmol/L. Unfortunately, healthy endemic control levels were not available in this study. No studies could be identified that investigate endemic healthy control levels in Kenya and Sudan, but a recent study in Ethiopia found a healthy control level of 3.8 nmol/L (IQR, 1.6–5.5 nmol/L) (Skogmar et al., 2015), which is in line with established average healthy control levels in European adults and children of 6.78 and 5.34 nmol/L for adults and children, respectively (Werner et al., 1987). Lingering immune activation could be a potential explanation for our observation, as was previously observed for HIV patients treated for 3–13 months with zidovudine or didanosine: neopterin concentrations remained elevated at approximately 19 nmol/L (Gisslen et al., 1997).

Currently there are no biomarkers either to identify treated VL patients at risk of relapse, or to establish final cure during the follow-up in clinical trials. This lack of early markers or test of cure is impeding the development of new antileishmanial treatment regimens. This study is the first evaluation of neopterin as a predictor of relapse in VL patients.

In conclusion, the identified 1.08 D60/EoT ratio cut-off—an >8% neopterin concentration increase between end of treatment and day 60—could serve as a surrogate endpoint identifying the patients in clinical trials who have an increased risk of relapse. Identified at-risk patients could be more intensively followed up in clinical trials, possibly using qPCR to quantify parasite loads in the blood and/or tissue to enable early detection of parasite recrudescence. The use of this neopterin parameter as a predictive biomarker for relapse in VL should be formally evaluated in a prospective trial, possibly in a panel of biomarkers to increase specificity.

## Author contributions

MW, AM, EK, FA, and TD were responsible for the clinical study conception and design. MW, AM, and EK were responsible for acquisition of the clinical data and samples. JB and JS provided the laboratory setting to perform the analysis. AK performed the laboratory analysis. AK and TD performed the data analysis and interpretation of the results. AK took the lead in writing the manuscript. All authors provided critical feedback and helped shape the research, analysis, and manuscript.

### Conflict of interest statement

The authors declare that the research was conducted in the absence of any commercial or financial relationships that could be construed as a potential conflict of interest.
